# TNF-α and IL-1α but not MCP-1 and Rantes increase significantly the formation of p-H2AX foci in naïve BM-derived TNFR1/p55KO EPCs

**DOI:** 10.1093/jrr/rrt199

**Published:** 2014-03

**Authors:** Sharath P. Sasi, Jin Song, Heiko Enderling, Xinhua Yan, David A. Goukassian

**Affiliations:** 1 CardioVascular Research Center, GeneSys Research Institute, Boston, MA, USA; 2 Center of Cancer Systems Biology, GeneSys Research Institute, Boston, MA, USA; 3 Tufts University School of Medicine, Boston, MA, USA

**Keywords:** TNF, TNFR2/p75, BM-EPCs, non-targeted, IL-1α

## Abstract

Background: Tumor necrosis factor-α (TNF) binds two receptors TNFR1/p55 and TNFR2/p75 and activates several signaling cascades. Ionizing radiation (IR) increases tissue levels of TNF. TNF signaling regulates numerous cytokines and chemokines that are known to mediate radiation-induced non-targeted effects (NTEs), a phenomenon where cells that are not directly ‘hit’ by IR exhibit IR effects as a result of signals received from nearby or distant IR cells. Little is known about the role of p55 or p75 in regulating NTE in bone marrow (BM) cells, specifically in BM-derived endothelial progenitor cells (EPCs). In media transfer experiments, we have previously shown that compared with WT EPCs, early NTEs (within 1–5 h) are inhibited in p55KO and p75KO EPCs, whereas delayed NTEs (within 3–5 days) are amplified in p55KO and to a lesser degree in p75KO EPCs, suggesting significant role of TNFR/p75 signaling (the remaining active receptor in p55KO EPCs) in mediating delayed NTEs. We hypothesized that signaling through TNFR2/p75 may alter radiation-induced TNF-mediated inflammatory response increasing tissue levels of various cytokines, chemokines and growth factors that could then induce NTE, possibly, via activation of NFkB and other stress response transcription factors.

Methods: To test our hypothesis *ex vivo*, expanded p55KO EPCs were irradiated with 1 Gy of γ-IR, then IR-conditioned medium (CM) was collected at 1, 5, 24 h, and 3, 5 days post-IR. CM from IR p55KO EPCs were processed for multiplex ELISA (12 proteins). After determining concentrations of each of 12 proteins in control and IR-CM media of p55KO EPCs over 5 days, we treated naïve p55KO EPCs with various concentrations of four mouse recombinant (rm) proteins that were steadily increased in IR-CM between Days 3–5. After 24 h incubation, naïve p55KO EPCs were stained with anti-p-H2AX antibodies and the formation of p-H2AX foci was visualized at ×100 magnification using laser scanning confocal microscopy. The p-H2AX foci were quantified manually by a single investigator blindfolded to the treatment conditions and were confirmed using computer-assisted algorithm.

Results: ELISA profiling of 12 proteins in IR-CM over 5 days post-IR showed 200–1600% increases (*P* < 0.02, at least, p55KO vs WT, Days 3–5) in cumulative levels of TNF, IFNr, IL1α, IL1β, IL6, EGF, MIP-1α, MCP-1, GCSF, GM-CSF, Rantes and Leptin. The steadiest and the highest increases between Days 3 and 5 were observed in IL-1α, MCP-1 and Rantes.

Naïve p55KO EPCs were then treated *ex vivo* with concentrations determined in the ELISA: IL-1α (290, 580 pg/ml), MCP-1 (580, 1160, 2900 pg/ml), Rantes (600, 1500 pg/ml) and TNF (100 pg/ml, 1, 40 ng/ml). After 24 h incubation with rm proteins, p55KO EPCs were stained with anti-p-H2AX antibodies. The cells were imaged and the quantification of the p-H2AX foci was performed as described above.

Results showed that the mean p-H2AX foci count of MCP-1 and Rantes was not significantly different from control which had a mean of 0.98 p-H2AX foci/cell count, with the exception of MCP-1 at 1160 pg/ml (*P* < 0.03, mean foci count of 1.9). TNF-treated naïve p55KO EPCs showed a significant increase in the mean p-H2AX foci/cell count at all concentration compared with the control, MCP-1 and Rantes (*P* < 0.0001 with the mean p-H2AX foci ranging from 2.8 to 3.9).

IL1α-treated p55KO EPCs showed the greatest increase in p-H2AX foci with the mean foci count of 7.1 at 290 pg/ml and 9.3 at 580 pg/ml, and was significantly different from all tested mouse recombinant proteins. Analysis of p-H2AX foci distribution of EPCs with one or more foci showed that in control p55KO EPCs, <1% of cells had a maximum of 4–9 p-H2AX foci/cell. Whereas in TNF- and IL-1α-treated p55KO EPCs, >2% and >4% of cells had 9–18 foci/cell, respectively. Remarkably, 1% of cells had as many as 18–31 foci/cell for TNF-treated cells and as many as 19–51 foci/cell for IL-1α-treated p55KO EPCs.

We conclude that TNF-TNFR2/p75 axis may induce NTEs in naïve BM-EPCs and suggest that blocking/neutralizing TNFR2/p75 signaling could represent a mitigating measure for prevention of delayed NTEs, specifically, in BM-derived EPCs and, conceivably, in BM milieu in general.

